# Impact of cancer on mortality and severity of corona virus disease 2019: A protocol for systematic review and meta-analysis

**DOI:** 10.1097/MD.0000000000023005

**Published:** 2020-10-30

**Authors:** Yi Zhang, Hao Han, Yunling Tian, Jing Dong, Yage Yu, Yingying Kang, Lina Xing, Rongna Lian, Ruinian Zhang, Dairong Xie

**Affiliations:** a Department of Geriatric Respiratory Medicine, Lanzhou University First Hospital; b Department of Critical Care Medicine, The Second Provincial People's Hospital of Gansu; c Department of Endocrinology, Lanzhou University First Hospital; d School of Basic Medical Sciences, Lanzhou University; e The First Clinical Medical College of Lanzhou University, Lanzhou, China.

**Keywords:** corona virus disease 2019, meta-analysis, mortality, neoplasms, severity

## Abstract

**Background:**

Cancer patients are in a state of systemic immunosuppression and are considered a highly vulnerable population in the Corona Virus Disease 2019 (COVID-19) epidemic. However, the relationship between cancer and the severity and mortality of patients with COVID-19 remains unclear. This study aims to evaluate whether cancer patients with COVID-19 may be at an increased risk of severe illness and mortality.

**Methods:**

We will perform comprehensive searches in PubMed, EMBASE.com, Web of Science, and the Cochrane Central Register of Controlled Trials to identify studies providing prevalence of cancer between patients with severe and non-severe illness or between non-survivors and survivors. We will use the Newcastle-Ottawa quality assessment scale to assess the quality of included studies. We will conduct pairwise meta-analyses to compute the odds ratio and 95% confidence interval using the Mantel Haenszel method with the random-effects model. The statistical heterogeneity will be assessed using the I^2^ statistic. Subgroup analyses, sensitivity analyses, and meta-regression analyses will be performed to explore the sources of heterogeneity.

**Results:**

The results of this study will be published in a peer-reviewed journal.

**Conclusion:**

Our meta-analysis will systematically evaluate the association between cancer and the severity and mortality of patients with COVID-19. This study will provide evidence to help determine whether cancer patients should be provided with special precautions and advised to use stronger personal protection.

**INPLASY registration number:**

INPLASY202090093.

## 1 Introduction

As of September 18, 2020, a total of 30,055,710 laboratory-confirmed Coronavirus disease 2019 (COVID-19) cases were reported worldwide, with 943,433 deaths (mortality rate: 3.1%).^[1]^ Several studies have evaluated the impact of comorbidities on patients infected with COVID-19, which showed that comorbidities in COVID-19 patients are associated with poor prognosis, including cerebrovascular disease, hypertension, obesity, chronic obstructive pulmonary disease, stroke, diabetes, cardiovascular disease, and kidney injury.^[2–8]^

The development of cancer is usually related to a blunted immune status,^[9,10]^ and anti-cancer treatments (such as chemotherapy) can also put cancer patients in an immunosuppressive state.^[11]^ Therefore, immunodeficiency may make cancer patients susceptible to COVID-19. Primary studies showed that cancer patients infected with COVID-19 had a higher risk of serious clinical events and death than those without cancer.^[12,13]^ A previous meta-analysis assessed the relationship between cancer and COVID-19 prognosis and found that there was no correlation between cancer and the severity of patients with COVID-19.^[14]^ However, another 2 meta-analyses suggested that the presence of cancer in COVID-19 patients increased the risk of developing serious complications.^[15,16]^ These meta-analyses did not reach consistent results. Furthermore, existing meta-analyses are limited by the small sample size and the conclusions are inconclusive. Therefore, a comprehensive meta-analysis is urgently needed to answer clinical questions. The primary objective of this study is to investigate the association between cancer and the severity of COVID-19. The secondary objective is to evaluate the association between cancer and COVID-19 mortality.

## 2 Methods

We will report this systematic review following the Preferred Reporting Items for Systematic Reviews and Meta-Analyses statement.^[17]^ This study protocol has been registered in the International Platform of Registered Systematic Review and Meta-Analysis Protocols (INPLASY, INPLASY202090093, doi: 10.37766/inplasy2020.9.0093).

### 2.1 Eligibility criteria

We will include studies that meet the following criteria:

(1) patients have a laboratory-confirmed diagnosis of COVID-19;
(2) provided the prevalence of cancer between COVID-19 patients with severe and non-severe illness or between non-survivors and survivors;
(3) with a sample size of larger than 10 patients;
(4) published in Chinese or English.

The following studies will be excluded:

(1) studies did not report data related to cancer patients;
(2) studies focused on only suspected cases or suspected cases and confirmed cases;
(3) review articles, protocols, guidelines, consensus, comments, abstracts, letters, and editorials.

### 2.2 Information sources and search strategy

We will perform comprehensive searches in PubMed, EMBASE.com, Web of Science, and the Cochrane Central Register of Controlled Trials (CENTRAL) up to September 30, 2020. We will use search terms include the following words: “severe acute respiratory syndrome coronavirus 2,” “ SARS-CoV-2,” “coronavirus disease-19,” “COVID-19,” “novel corona virus,” “new coronavirus,” “2019-nCoV,” “novel coronavirus,” “nCoV-2019,” “novel coronavirus pneumonia,” “2019 novel coronavirus,” “coronavirus disease 2019,” “neoplasms,” “neoplasia,” “tumor,” “tumour,” “cancer,” “malignancy,” “clinical characteristic,” “clinical feature,” “risk factor,” “prognosis,” and “comorbidities.” The search strategy of PubMed is presented in Table 1. Reference lists of eligible studies and relevant systematic reviews will be also manually searched for potentially eligible studies.

**Table 1:**
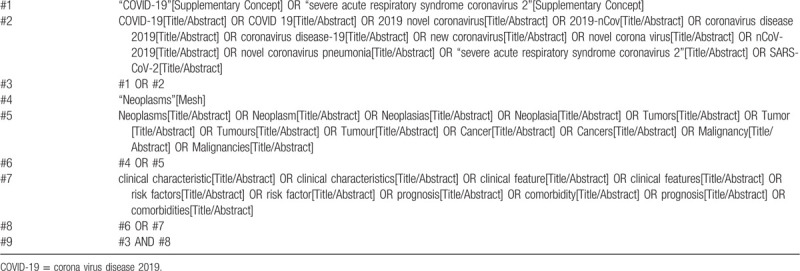
Search strategy of PubMed.

### 2.3 Study selection process

We will import the retrieved records into EndNote X8 (Thomson Reuters (Scientific) LLC Philadelphia, PA) software for management. Two authors independently will screen the titles and abstracts of the records to determine if they meet the inclusion criteria. Then, the same 2 authors will retrieve the full text of all potentially eligible studies and assess the eligibility of each study according to the inclusion criteria. Regarding multiple studies from the same teams or studies with samples from the same settings, we will evaluate the time frame and detailed data of the study. For studies with overlapping data, we will include the study with a larger sample size. Conflicts will be resolved by discussions with a third reviewer.

### 2.4 Data extraction

We will develop a standardized data extraction form using Microsoft Excel 2016 (Microsoft Corp, Redmond, WA, www.microsoft.com) through discussions with the review team and will revise it after piloting on a random of 5 studies. The data will be extracted include: study characteristics (first author, year of publication, country of the corresponding author, journal name, publication language, study setting, recruitment time frame), population characteristics (age, sex, sample size), and outcomes of interest (number of cancer patients, severe cases, non-severe cases, deaths, and survivors). The severe illness is defined in this study as patients experiencing acute respiratory distress syndrome, requiring mechanical ventilation, requiring vital life support, or requiring intensive care unit admission support.^[18,19]^ The data extraction will be performed by 1 reviewer and checked by a second reviewer. Discrepancies will be resolved by consensus or by the discussion with a third reviewer.

### 2.5 Risk of bias assessment

We will use the Newcastle-Ottawa quality assessment scale to assess the quality of included studies.^[20]^ Studies that obtained more than 7 stars will be considered as high quality, 5 to 7 stars will be considered as moderate quality, and lower than 5 stars will be considered as low quality.^[21]^ Two reviewers will conduct quality assessment independently. Any conflicts will be resolved by consensus or by the discussion with a third reviewer.

### 2.6 Statistical analysis

We will conduct pairwise meta-analyses to compute the odds ratio and 95% confidence interval of cancer prevalence in COVID-19 patients with or without severe illness, and non-survivors or survivors. The meta-analyses will be performed using the Mantel Haenszel method with the random-effects model. The statistical heterogeneity will be assessed with the *I*^2^ statistic, and value of < 25%, 26% to 50%, and > 50% will be considered as low, moderate, and high level of heterogeneity, respectively.^[22]^ All analyses will be conducted using Stata (13.0; Stata Corporation, College Station, Texas) and the statistical level of significance will be set at *P* < .05.

### 2.7 Subgroup analysis, sensitivity analysis, and meta-regression analysis

We plan to conduct subgroup analyses of the outcomes between different countries. Sensitivity analyses will be conducted by excluding studies with high risk of bias to assess the stability of results. We will also perform univariate meta-regression analyses to assess if either the outcomes or the heterogeneity is associated with the publication languages and number of centers of the study conducted.

### 2.8 Publication bias

The funnel plot and Egger test will be adopted to detect the publication bias for outcomes with studies no fewer than ten.

### 2.9 Certainty of evidence

The certainty of evidence for each meta-analysis will be evaluated using the Grading of Recommendations Assessment, Development and Evaluation framework, which rated the quality considering 5 criteria: risk of bias, inconsistency, imprecision, indirectness, and publication bias.^[23–25]^ The quality of evidence will be rated as high, moderate, low, or very low and the results will be presented in the Summary of Findings table.

## 3 Results

### 3.1 General characteristics of initially included studies

We conducted preliminary searches and included 5 studies^[26–30]^ to conduct a pilot analysis. All included studies were from China and published in English. The sample size per study ranged from 191 to 1590 and the total sample size was 2619. The detailed characteristics are summarized in Table 2.

**Table 2:**
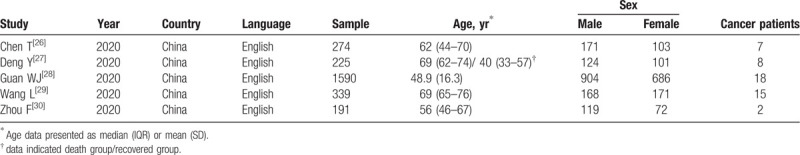
Characteristics of included studies.

### 3.2 Association between cancer and COVID-19 mortality

**Figure 1:**
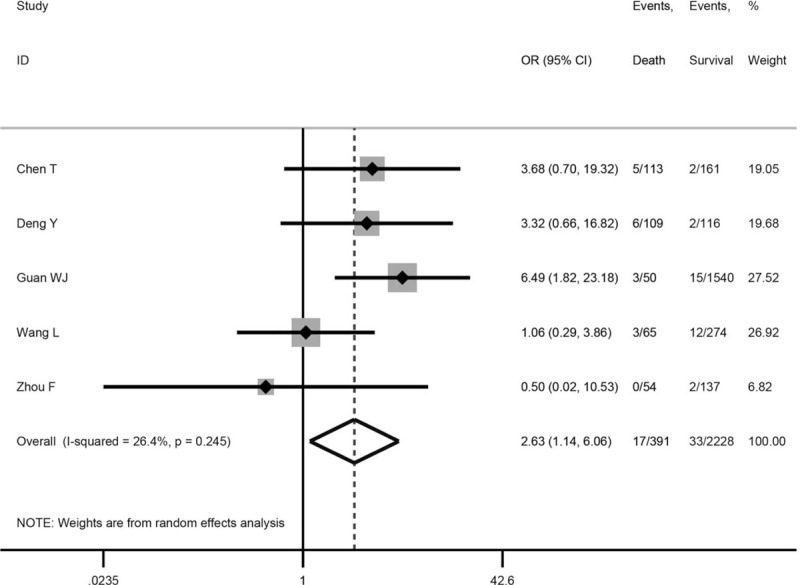
Association between cancer and the mortality of COVID-19. COVID-19 = corona virus disease 2019.

Five studies^[26–30]^ involving 2619 patients provided cancer prevalence with comparison between dead and surviving COVID-19 patients. The meta-analysis revealed that cancer was associated with a significantly enhanced risk of death (odds ratio = 2.63, 95% confidence interval: 1.14 to 6.06, *P* = .023; *I*^2^ = 26.4%) (Fig. 1).

## 4 Discussion

Cancer patients are in a state of systemic immunosuppression and are considered a highly vulnerable population in the COVID-19 epidemic.^[11,12]^ Our meta-analysis will systematically evaluate the association between cancer and the severity and mortality of patients with COVID-19. Our study will provide evidence to help determine whether cancer patients should be provided with special precautions and advised to use stronger personal protection. We hope the results of our meta-analysis can also provide the latest references for the development of new guidelines.

## Author contributions

YZ, HH, YLT, JD, YGY, YYK, LNX, RNL, RNZ, and DRX planned and designed the research. YZ, JD, YGY, YYK, LNX, RNL, RNZ, and DRX tested the feasibility of the study. HH and YLT provided methodological advice and revised the manuscript. YZ and HH wrote the manuscript. All authors approved the final version of the manuscript.

**Conceptualization:** Yi Zhang, Hao Han.

**Formal analysis:** Yi Zhang, Yunling Tian.

**Funding acquisition:** Yi Zhang.

**Investigation:** Yunling Tian, Jing Dong, Yage Yu, Yingying Kang, Rongna Lian, Ruinian Zhang, Dairong Xie.

**Methodology:** Yi Zhang, Jing Dong.

**Project administration:** Yi Zhang.

**Resources:** Yi Zhang, Yunling Tian, Jing Dong, Yage Yu, Yingying Kang, Lina Xing.

**Supervision:** Hao Han.

**Visualization:** Yunling Tian, Jing Dong, Yage Yu, Lina Xing, Rongna Lian, Ruinian Zhang, Dairong Xie.

**Writing – original draft:** Yi Zhang, Hao Han.

**Writing – review & editing:** Yi Zhang, Hao Han.
